# Design and study of anticaries effect of different medicinal plants against *S.mutans glucosyltransferase*

**DOI:** 10.1186/s12906-019-2608-3

**Published:** 2019-08-02

**Authors:** Kiranmai Mandava, Uma Rajeswari Batchu, Shravya Kakulavaram, Shulamithi Repally, Ishwarya Chennuri, Srinivas Bedarakota, Namratha Sunkara

**Affiliations:** 1 Department of Pharmaceutical Chemistry, Bharat Institute of Technology, Mangalpally, JNTUH, R.R. District, Hyderabad, Telangana 501510 India; 2 Department of Pharmaceutical Biotechnology, Bharat Institute of Technology, Mangalpally, JNTUH, R.R. District, Hyderabad, 501510 India

**Keywords:** *S.mutans*, Glucosyltransferase, *Azadirachta indica*, *Terminalia chebula*, Polyherbal mouth wash, Anticaries agent

## Abstract

**Background:**

The present study was aimed to evaluate the molecular level anticaries effect of different medicinal plants against *Streptococcus mutans* (*S.mutans)* glucosyltransferases (gtf).

**Methods:**

A total of six natural sources named as *Terminalia chebula (T.chebula), Psidium guajava (P.guajava), Azadirachta indica (A.indica) and Pongamia pinnata (P.pinnata)*; two essential oils, clove (*Syzygium aromaticum*) and peppermint oil (*Mentha piperita*) were selected as test samples. Hydroalcoholic plant extracts and essential oils were examined for their inhibitory potential on *gtf* isolated from *S.mutans.* Polyherbal mouth wash was prepared and its effect on *gtf* activity was compared with commercial chlorhexidine mouth wash (5%w/v). Enzyme kinetic study was carried out in order to explore the molecular mechanism of enzyme action.

**Results:**

Out of six natural sources tested, *A.indica* has shown maximum inhibitory effect of 91.647% on *gtf* and *T.chebula* has shown IC_50_ of 1.091 mg/ml which is significant when compared to standard chlorhexidine. From the final result of kinetic analysis it was found that *T.chebula, P.guajava* and *P.pinnata* have show uncompetitive inhibition where as *A.indica has shown non-competitive inhibition.* Surprisingly, both essential oils have shown allosteric inhibition (sigmoidal response). The polyherbal moutwash has shown significant inhibitory potential on *gtf* (95.936%) when compared to commercial chlorhexidine mouthwash (*p* < 0.05).

**Conclusion:**

All the tested samples have shown considerable *gtf* inhibitory action. Moreover polyherbal mouth wash has shown promising noncompetitive inhibitory activity against *gtf* and it could be the future formulation to combat dental caries.

## Introduction

Fermentation of carbohydrates presents in a food by oral bacteria results in a decrease in the pH of plaque and demineralization of enamel and finally formation of dental caries [1–4]. *Streptococcus mutans* (*S.mutans*) is considered to be the principal cariogenic bacterium in humans [5, 6]. It possesses several virulence factors that are associated with its cariogenicity [7]. An essential factor of *S.mutans* is its sucrose-dependent and glucan-mediated colonization of tooth surfaces. Glucans are glucose polymers synthesized by extracellular glucosyltransferases (gtfs) [1]. There are three *S.mutans* gtf’s: gtf B and gtf C synthesize primarily water-insoluble glucans (WIG) [8, 9] and gtf D, synthesize water-soluble glucans (WSG) [10]. Gtfs are involved in many biological processes such as cell-cell communications, signal transduction, immune response, microbial adhesion and infection [11]. Biofilm or dental plaque is formed through two different sequential steps: initial and reversible cell-to-surface attachment and subsequent sucrose-dependent adhesion of the microorganisms, which is firm and irreversible, gtfs are strongly involved in the latter step [12]**.** There are multiple ways that compounds can exert anticaries action in addition to gtf inhibition. In view of essential roles played by gtfs, intervention of gtfs has attracted remarkable interest among other ways for drug development since inhibitors of gtfs can potentially interfere with the pathological processes [13, 14]. Targeting gtf enzymes prevents the synthesis of extracellular polysaccharides (glucans) and is an attractive strategy for the development of anti-biofilm compounds, as the glucans are extremely important in the processes of biofilm formation and stabilization [15].

The use of medicinal plants or natural products has been one of the most successful strategies for the discovery of new medicines [16]. It already been reported that plant extracts and their components have significant antibacterial activity on oral bacteria, especially on *S.mutans* [17–21]*.* Phytochemical rich extracts and their associated compounds have repeatedly shown inhibitory effects against adhesion, plaque and biofilm formation of *S.mutans* [22–27]*.* As inhibition of essential virulence factor (gtf) is the primary goal for the prevention of dental carries and possibly other plaque related diseases, present study is aimed to report the anti-caries effects of four plant extracts (Gallnut, Guava, Neem, Indian beech) and two essential oils (Clove oil, Peppermint oil) against *S.mutans* gtfs.

## Material and methods

Brain Heart Infusion broth (BHI), sucrose, ethanol, sodium dodecyl sulfate, acetic acid, sodium acetate, tween 80, dimethyl formamide (DMF) and ammonium sulfate (all are of analytical grade) were used in the present study and purchased from Merck, Mumbai.

### Design of the study

Four medicinal flora, *Terminalia chebula (T.chebula), Psidium guajava (P.guajava), Azadirachta indica (A.indica) and Pongamia pinnata (P.pinnata)*; two essential oils, clove (*Syzygium aromaticum*) and peppermint oil (*Mentha piperita*) were selected as test samples for the present study. By keeping in survey of the folklore use of these plants in dentistry and their reported antibacterial activity [28–31], specific parts of the above mentioned plants like fruits of *T.chebula*, leaves of *P.guajava*, twigs of *A.indica* and *P.pinnata* were selected. In a similar way essential oils of clove and peppermint were selected as essential oils have stood out as a promising source of bioactive molecules with potential application in the management of dental caries [32, 33].

### Collection and authentication of plant material

Fruits of *T. chebula (068)* were purchased from local commercial market. The leaves of *P. guajava (081),* twigs of *A. indica (0125)* and *P. pinnata (001)* were collected from the institutional medicinal garden (cultivated), Bharat Institute of Technology, Hyderabad. All the collected plant materials were authenticated by Department of Botany, Osmania University, Hyderabad and voucher specimens were submitted in the same place. Clove oil and peppermint oil were procured from essential oil manufacturer (PSC aromatics), Ooty, Tamilnadu.

### Preparation of hydroalcoholic extracts of plants

Plant materials were shade dried and pulverized separately. Each plant material was extracted separately using an equimolar ratio of ethanol and water (50:50) by using a soxhlet extraction method. The hydroalcoholic extracts were concentrated using a rotary evaporator and the dried extracts were preserved at 4 °C for further use.

### Culture collection

*S.mutans MTCC 49* strain used for the present study was facultatively anaerobic, Gram-positive coccus-shaped bacterium, procured from the centre for cellular and molecular biology (CCMB), Tarnaka, Hyderabad. A stock of *S.mutans MTCC 49* was prepared in glycerol and preserved at 0 °C until further use.

### Production and partial purification of gtf

A partially purified gtf required for enzyme inhibition studies was prepared according to the modified method of Tomita *et.al* [34]. BHI broth containing 5% sucrose was prepared and *S.mutans* MTCC 49 were inoculated into the media. Furthermore tween 80 (1 mg/ml) was incorporated into the media to increase the enzyme production [35]. The inoculated broth was then incubated for 24 h at 37 °C. Bacteria grown in 600 ml BHI broth were centrifuged (1800Xg, 30 min) and supernatant fluid obtained was precipitated using 2/3 volume of ammonium sulfate (60%). The precipitate was centrifuged and dissolved in 30 ml phosphate buffer (pH 7.4) and dialyzed for 24 h against the same solution. The insoluble material was removed by centrifugation. The dialysate was preserved at 0 °C until further use.

### Determination of cell free gtf activity

Gtf activity was determined by modified methods of Fukushima *et.al* [36].

### Assay for WIG forming activity (method 1)

The gtf activity was determined based on the amount of glucans formed, expressed as the absorbance at 340 nm (ΔA_340_/min). The reaction mixture composed of 100 μM citrate buffer (pH 6.2), 5% sucrose, phosphate buffer of pH 7.4 and 0.5 ml of partially purified gtf extract was incubated at 25 °C for 10 min. The increase in absorbance at 340 nm was determined using UV-VIS spectrophotometer (Shimadzu 1800). The activity (ΔA_340_/min) of gtfs was determined from the slope of the linear part of the time course curve.

### Calorimetric assay for WIG and WSG forming activity (method 2)

In this method gtf activity was determined based on the amount of glucans formed, expressed as the glucose content per minute. The reaction mixture composed of 100 μM citrate buffer (pH 6.2), 5% sucrose, phosphate buffer of pH 7.4 (control), 0.5 ml of partially purified gtf extract was incubated at 37 °C for 2 h. The reaction was terminated by the addition of 0.6 M sodium dodecyl sulfate. WIG pellet was precipitated by centrifugation at 10,000×g for 5 min. The supernatant was then transferred to another tube and WSG was precipitated with 3 volumes of ethanol at 4 °C over night. The washed polysaccharide (WIG & WSG) was quantified based on the glucose content using phenol-sulfuric acid method [37]. For the quantification of WIG and WSG, 50 μl of the sample was used in the assay.

### Determination of the effect of plant extracts on gtf activity

The gtf inhibition studies using different concentrations of plant extracts were carried out by method 1. Plant extracts were dissolved in phosphate buffer with gentle heating where necessary and diluted to give final concentrations of 0.312, 0.625, 1.25, 2.5, 5 and 10 mg/ml in the reaction mixture. The reaction mixture used in the method 1 was incubated replacing phosphate buffer with various concentrations of the plant extracts for 10 min at 25 °C. The amount of glucan formed was determined according to the procedure described in method 1. To adjust for the quantification errors due to carbohydrates of plant extracts which will precipitate with the glucans, a parallel series of mixtures without the enzyme was prepared and absorbance value was subtracted from test assay readings.

### Determination of the effect of essential oils on gtf activity

The effect of essential oils on gtf was determined by method 2 to overcome the solubility problem of essential oils with enzyme solution in method 1. The two essential oils used in the present study were diluted with DMF separately to make concentrations of 3.125, 6.25, 12.5, 25, 50 and 100 mg/ml. The reaction mixture used in the method 2 was incubated with various concentrations of the test oils for 2 h at 37 °C. The amount of WIG and WSG was estimated by the method 2 described above.

### Formulation of poly herbal mouth rinse [38]

The poly herbal mouth rinse formulation was designed based on IC_50_ values obtained for each plant extract and essential oils (Table 1). It was evaluated for pH and clarity test. Finally the effect of mouth rinse against gtf activity was determined and compared with commercial chlorhexidine mouthwash.

**Table 1:** Formulation of polyherbal mouth rinse

Ingredients	Working formula
*T. chebula*	1.091 w/v
*A. indica*	9.262 w/v
*P. guajava*	5.099 w/v
*P. pinnata*	5.63 w/v
*Clove oil*	63.697 w/v
*Peppermint oil*	44.693 w/v
*Ethanol*	6 v/v
Tween 80	3.5 v/v
Distilled water	upto 50 ml

### Enzyme kinetics

For kinetic analysis K_M,_ Vmax and hill coefficient values were measured with non-linear regression analysis using Graphpad Prism 5.0 version.

### Statistical analysis

All the experiments were carried out in triplicates and data was presented as mean ± SEM. Statistical analysis and enzyme kinetics were performed using Graph pad Prism 5.0 software.

## Results and discussion

The percentage yield of hydro alcoholic extract of *T.chebula, P.guajava, A.indica and P.pinnata* was found to be 0.480, 0.27, 0.87 and 1.177%w/w respectively.

Dental caries is a biofilm-mediated, sugar driven, multifactorial, dynamic disease that results in the phases of demineralization and remineralization of dental hard tissues. The synthesis of extracellular polysaccharide, including WIG & WSG is one of the most important virulence factors of *S.mutans* [39]***.*** The WIG promotes the adhesive interactions of bacteria with the tooth surfaces and contributes the formation of dental biofilm [40]**.** Accordingly, in the present study, we examined whether selected plant extracts inhibits the synthesis of WIG and whether clove and peppermint oils inhibits the synthesis of WSG & WIG by crude gtfs as there is no previous molecular level mechanistic report on these samples. Our results were in correlation with the literature reports of the anti *S.mutans* activity of the test samples.

**Fig. 1:**
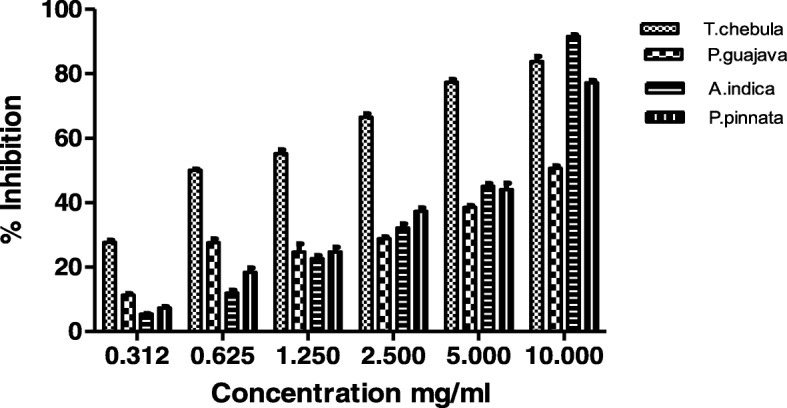
Glucosultransferase inhibitory effects of T.chebula, P.guajava, A.indica and P.pinnata

The hydroalcoholic extracts of *T.chebula, P.guajava, A.indica and P.pinnata* inhibit WIG synthesis by inhibiting *S.mutans* gtf activity in a dose dependent manner (Table 2). *A.indica* extract has shown highest percentage inhibition of 91.647 ± 0.445% at 10 mg/ml concentration. Order of gtfs inhibitory activity of the four plant extracts at their highest concentration of 10 mg/ml was *A.indica > T.chebula > P.pinnata > P.guajava. T.chebula* extract has shown predominant inhibitory action among tested extracts from 0.312 to 5 mg/ml (Fig. 1). It means gtf synthesizing WIG from sucrose was almost suppressed upon addition of 10 mg/ml concentration of *A.indica* extract. Highest correlation coefficient (R^2^) value is observed with *A.indica* when compared to other extracts. However, according to the 50 % inhibitory doses (IC_50_) *T.chebula* would be considered as an effective gtf inhibitor as it has shown effect at lower concentration (1.091 mg/ml) when compared to other three plant extracts. It has been reported that gtf B and C are important factor for synthesis of WIG by *S.mutans,* gtf B synthesize primary WIG where as gtf C synthesizes WIG & WSG [41]**.** Although we identified that *T.chebula, P.guajava, A.indica and P.pinnata* extracts may inhibits gtf B by inhibiting WIG synthesis where as clove and peppermint oil inhibits both gtf B and gtf C by inhibiting WIG & WSG synthesis.

**Table 2:** Gtf inhibitory effect of different plant extracts at various concentrations

Concentration mg/ml	T.chebula	P.guajava	A.indica	P.pinnata
0.312	27.667 ± 0.664*	11.300 ± 0.529	5.373 ± 0.202	7.317 ± 0.368
0.625	50.047 ± 0.307*	27.533 ± 1.210	11.887 ± 0.873	18.403 ± 1.291
1.25	55.200 ± 1.159*	24.610 ± 2.623	22.667 ± 0.841	24.403 ± 1.444
2.5	66.530 ± 1.136*	28.817 ± 0.592	32.143 ± 1.206	37.260 ± 1.155
5.0	77.367 ± 0.939*	38.560 ± 0.544	45.067 ± 0.902	44 ± 2.031
10	83.820 ± 1.615	50.593 ± 0.903	91.647 ± 0.445*	77.173 ± 0.817
R^2^	0.9673	0.8161	0.9700	0.9412
F-value	9.487	22.55	248	82.29
IC50 mg/ml	1.091	9.262	5.099	5.63

Values are expressed in mean ± SEM and data were analyzed by one way ANOVA at *p < 0.05

**Fig. 2:**
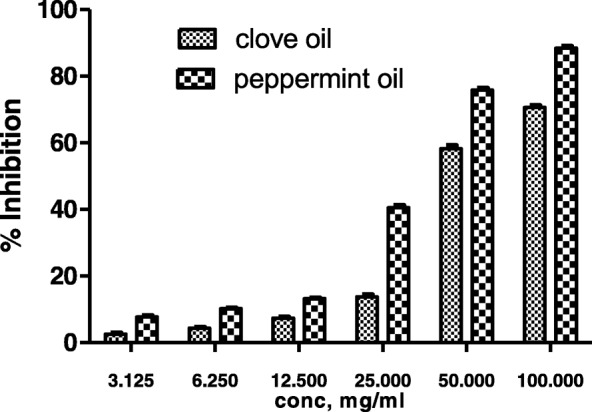
Gtf inhibitory effects of clove and peppermint oils at various concentrations

**Fig. 3:**
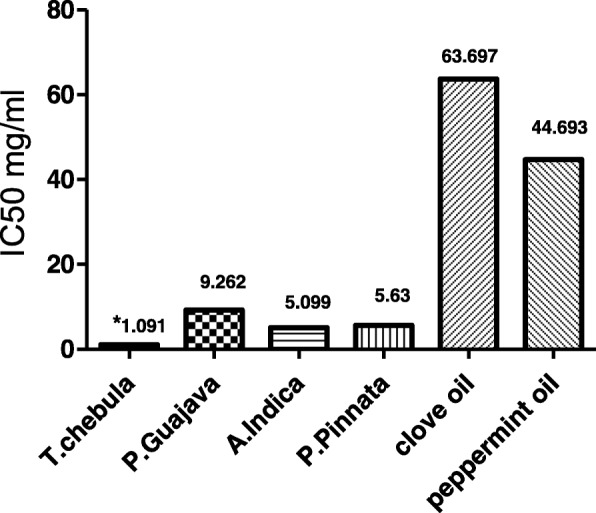
IC50 values of plant extracts and essential oils

To study the molecular level mechanism responsible for the anticaries activity of clove oil and peppermint oil, the effect of these on the WIG & WSG synthesis by cell free extracellular gtf of *S.mutans* were examined. Various concentrations ranging from 3.125-100 mg/ml were used for both the samples and their concentrations are different from that of concentrations used for plant extracts (based on previously reported MIC against *S.mutans*). Dose dependent inhibition of gtf was observed in both the cases (Table 3) and especially from 25 to 100 mg/ml there was a drastic increase in % inhibition. IC_50_ values of peppermint oil (44.693%) was significant when compared to clove oil (63.697%) (Fig. 2). Out of six test samples used in the present study, *T.chebula* extract has shown the low IC_50_ value (Fig. 3).

**Table 3:** Gtf inhibitory effect of clove and peppermint oils at various concentrations

Concentration, mg/ml	Clove oil	Peppermint oil
3.125	2.583 ± 0.306	7.717 ± 0.4234
6.250	4.402 ± 0.234	10.227 ± 0.127
12.500	7.373 ± 0.320	13.193 ± 0.156
25.000	13.8 ± 0.577	40.567 ± 0.606
50.000	58.267 ± 0.960	75.867 ± 0.606
100.000	70.667 ± 0.521	88.483 ± 0.563
R^2^	0.898	0.879
IC_50_	63.697	44.693
F	140.8	117.1

Values are expressed in mean ± SEM

R^2^ value represents square root of the correlation coeffecient and *A.indica* has R^2^ value of 0.9700 indicates 0.9700^2^ × 100 (97%), the higher the percentage better the correlation between results. F-value is a numerical indicator of whether or not the result expressed by the method is coincidental, the higher the value, the less likely the result is due to chance (*A.indica* has highest F-value of 248 among tested extracts).

**Fig. 4:**
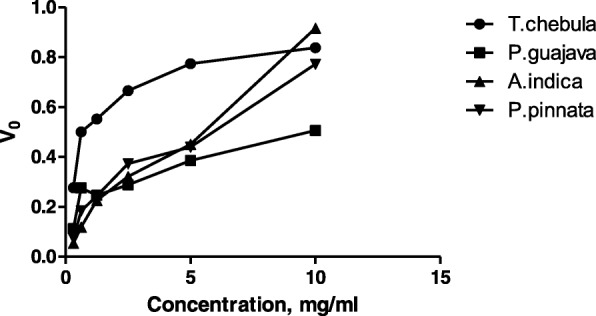
Exponential response curves of *T.chebula, P.guajava, A.indica and P.pinnata* extracts

From kinetic analysis it was observed that *T.chebula, P.guajava, A.indica and P.pinnata* have shown the exponential curves (Fig. 4) indicative of deviation from normal response (hyperbolic) of the enzyme. From their Michael Menton constant (K_M_) and Vmax values, it was identified that *T.chebula, P.guajava* and *P.pinnata* have shown uncompetitive inhibition as their K_M_ and Vmax values have been decreased when compared to control (without inhibitor). In case of *A.indica* it was found that K_M_ unchanged and Vmax decreased indicated that the pattern was of non-competitive inhibition (Table 4).

**Table 4:** Kinetic parameters of the inhibitory effect of test samples on the activity of *gtf*

	Control	T.chebula	P.guajava	A.indica	P.pinnata	Clove oil	Peppermint oil
K_M_	21.30	0.6115	1.006	20.82	5.101	211.20	73.64
V_max_	25.9	3.695	6.940	13.74	23.16	231.21	161.41
Hill coefficient	NA	NA	NA	NA	NA	4.60 ± 0.59	1.92 ± 0.46

**Fig. 5:**
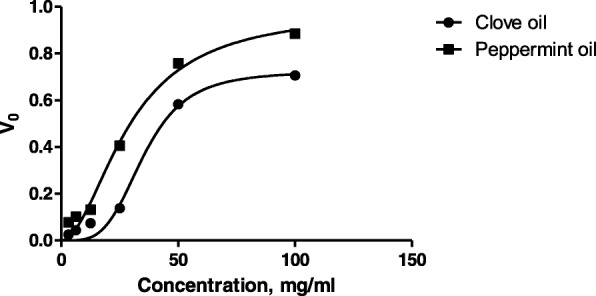
Sigmoidal response curves of clove oil and peppermint oil

Similar analyses of clove and peppermint oils revealed the fact that response was sigmoidal and the pattern was of allosteric inhibition (Fig. 5). Sigmoid response curve suggests the cooperativity between the inhibitor and the enzyme and hill coefficient provides a way to quantify the degree of binding between inhibitor and enzyme. Both clove and peppermint oil have shown hill coefficient > 1 represents positive, cooperative binding (Table 4). Hill coefficient value is more (4.60 ± 0.59) for clove oil when compared to peppermint oil (1.92 ± 0.46) indicates that clove oil has more affinity for gtf. The present study mainly focused on the effect of plant extracts and essential oils on gtf inhibition and comparison of the same with commercial anti-carries agent. Here the criteria is to compare molecular level gtf inhibitory mechanism of tested samples so that whether they possess significant anti carries property or not.

The bilologically active phytoconstituents or secondary metabolites present in all four plant extracts have been responsible for their gtf inhibitory activity. Moreover gtf activity of bioactive compounds like tannins [42], flavonoids [43], terpenoids [44] were reported previously. The tannins in *T.chebula* fruits, terpenoids in *A.indica* twigs, isoflavones in twigs of *P.pinnata* and flavonoids and tannins present in leaves of *P.guajava* may be the attributing factors for *S.mutans* gtf inhibition**.**

The antibacterial properties of plant essential oils against bacteria found in the oral cavity are also well documented [45]. While the commercial mouthwashes containing essential oils are useful in the long-term control of plaque and mild to moderate gingivitis and are preferred to use in chlorhexidine containing mouth washes for long-term daily use [46]. Therefore, essential oils may be suitable additives in pharmaceutical products.

There has been reports about the menthol present in *Mentha piperata* (peppermint oil) and eugenol in *Syzygium aromaticum* (clove oil) and are considered as outstanding compounds exhibiting an antibacterial potential [47] which could be responsible for *S.mutans* gtf inhibition*.*

**Fig. 6:**
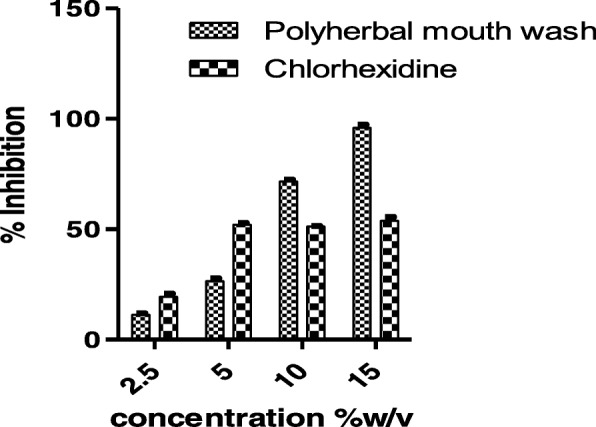
Gtf inhibitory effects of Polyherbal mouth rinse and Chlorhexidine mouth rinse at various concentrations

**Fig. 7:**
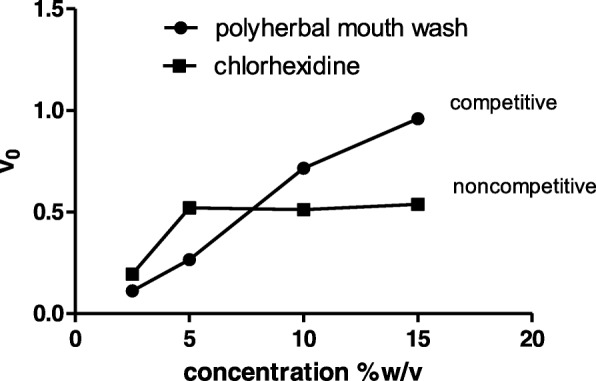
Exponential response curves of Polyherbal mouth rinse and Chlorhexidine mouth

The present study also focused on the formulation of the polyherbal mouth rinse and its IC_50_ value was found to be 7.938%w/v. The % inhibition at its maximum concentration of 15%w/v was found to be 95.936 ± 1.518 and it was very high when compared to 53.80 ± 1.83% inhibition of chlorohexidine commercial mouthwash. Dose dependent gtf inhibitory effect was observed in case of polyherbal mouth wash and chlorhexidine did not follow this pattern. From the results it was observed that gtf inhibitory potential of the polyherbal mouth rinse was significant (*p* < 0.05) compared to chlorhexidine mouthwash at tested concentrations (Fig. 6). From the shape of the exponential curve (concentration of inhibitor vs enzyme velocity) it was evident that polyherbal mouth rinse competitively inhibits gtf where as chlorhexidine shows non-competitive inhibition (Fig. 7).

## Conclusions

From the studies it was concluded that among the four plant extracts and two essential oils tested against gtf activity *A.indica* extract has shown maximum percentage inhibition and *T.chebula* extract has shown the significant IC_50_ value when compared to others. The results of kinetic analysis proposed that *T.chebula, P.guajava, P.pinn*ata have shown uncompetitive gtf inhibition where as *A.indica* has shown non-competitive inhibition. Clove oil and Peppermint oil have proposed allosteric inhibition and the standard chlorhexidine has shown non-competitive inhibition. Moreover the polyherbal mouth rinse prepared from all four plant extracts and two essential oils has shown predominant gtf inhibition (95.936%) when compared to 54% shown by chlorohexidine mouthwash. Further clinical investigations are recommended for this polyherbal mouth rinse to utilize as anticaries agent.

## Data Availability

All data and materials used in this research are available from the corresponding author on reasonable request.

## Abbreviations

*A.indica*: *Azadirachta indica*
BHI broth: Brain heart infusion broth
DMF: Dimethyl formamide
Gtfs: Glucosyltransferases
MIC: Minimum inhibitory concentration
*P.guajava*: *Psidium guajava*
*P.pinnata*: *Pongamia pinnata*
*S.mutans*: *streptococcus* mutans
*T. chebula*: *Terminalia chebula*
WIG: Water insoluble glucans
WSG: Water soluble glucans
